# ChatGPT or LLM in next-generation drug discovery and development: pharmaceutical and biotechnology companies can make use of the artificial intelligence-based device for a faster way of drug discovery and development

**DOI:** 10.1097/JS9.0000000000000719

**Published:** 2023-09-13

**Authors:** Soumen Pal, Manojit Bhattacharya, Md. Aminul Islam, Chiranjib Chakraborty

**Affiliations:** a School of Mechanical Engineering, Vellore Institute of Technology, Vellore, Tamil Nadu; b Department of Zoology, Fakir Mohan University, Vyasa Vihar, Balasore, Odisha; c Department of Biotechnology, School of Life Science and Biotechnology, Adamas University, Kolkata, West Bengal, India; d Advanced Molecular Lab, Department of Microbiology, President Abdul Hamid Medical College, Karimganj, Kishoreganj; e Department of Microbiology, COVID-19 Diagnostic Lab, Noakhali Science and Technology University, Noakhali, Bangladesh

*Dear Editor*,

Two recent correspondence articles published in this journal about artificial intelligence (AI) in drug discovery and development are timely. The first article by Chakraborty C. *et al*. discussed AI in drug discovery and development, and the second article by Chakraborty C. illustrated AI in clinical trials^1,2^. These two articles elucidate the readers about the recent trend of AI in drug discovery and development. Recently, the increased application of AI has been observed in various sectors of society, including the healthcare sector. AI has been used in healthcare, from disease diagnosis to therapeutic discovery. AI-based drug discovery has played a significant role as a whole process of drug discovery and development. These two articles also provide significant information on AI-enabled drug discovery and development. However, one of the significant milestones of AI-based discovery was achieved by two companies, Exscientia and Sumitomo Dainippon Pharma, through the collaboration. Their discovered drug has entered into the clinical trial. Exscientia is a British start-up, and Sumitomo Dainippon Pharma is a Japanese pharmaceutical company. The drug molecule was developed using sophisticated AI methodologies, and the name of the molecule was DSP-1181^3^. Recently, AI-enabled ChatGPT has shown its application in other areas of medicine and healthcare, including drug discovery and development.

ChatGPT was popularized very fast after its first introduction in November 2022. It has been noted that more than one million users have utilized the AI-enabled device within 5 days after its release^4^. The ChatGPT's is a state-of-the-art natural language processing system ingeniously developed by OpenAI in 2022^5^. This cutting-edge system is endowed with the remarkable ability to generate human-like conversations by adroitly comprehending a conversation’s context and producing appropriate responses. Using this characteristic, researchers and users have explored ChatGPT’s several medical applications. Similarly, the AI-enabled ChatGPT application has been used in drug discovery and development. Recently, an article published in Nature Biotechnology has highlighted that he ChatGPT or large language models (LLM) are helping scientists to discover new drug targets. The article describes that AI-driven ChatGPT can map several potential drug targets, which helps drug discovery researchers from pharmaceutical companies in faster methods of drug discovery^6^. Therefore, there are immense possibilities that ChatGPT or LLM can be used in the different steps of drug discoveries. In this direction, we illustrate the possible contribution of ChatGPT or LLM in next-generation drug discovery and development. It has been noted that discovering drugs and their development is an intricate and multifaceted endeavor encompassing many stages, from the initial identification of a potential therapeutic target to the rigorous testing and eventual regulatory approval of a successful drug candidate. In this direction, ChatGPT or LLM are helping to perform all these steps successfully.

The conventional drug discovery and development approach has been arduous, expensive, and time-consuming. The pharmaceutical industry invests billions of dollars in introducing a potential drug candidate into the market, and it typically takes 15–20 years of research to identify a novel drug candidate^7,8^. Several researchers calculated the drug development cost, and the investment for a new drug molecule was estimated from research and development to market at about $1141.7 million^9^. Traditional drug discovery faces challenges, including high failure rates, low efficiency, and high costs. The high failure rate is attributed to the inability to predict drug candidates’ efficacy and toxicity accurately. Low efficiency is due to the demanding and labor-intensive nature of the process, which requires synthesizing and testing many compounds. The high-cost results from the need for expensive equipment, facilities, and personnel. An additional essential constraint in the initial stages of drug exploration has been the absence of unanimity concerning a suitable in vivo experimental model that can effectively imitate human ailments^10^. Recently, Sharma and Thakur illustrated that AI-assisted ChatGPT performs various steps of drug discovery, such as calculating the compound multiplicity, file conversion input file generation for Gaussian software, searching the pdb files, input file generation for docking, etc. All these activities help develop highly effective drug molecules^11^. ChatGPT or LLMs have been able to perform the different trajectories towards the discovery to development. Its robust and sophisticated features have contributed immensely to drug discovery by providing many advantageous outcomes. Among these benefits is the ability to analyze high-content screening data and design and synthesize new molecules. LLM models have facilitated the prognostication of biological activities and ADME properties encompassing absorption, distribution, metabolism, and excretion. Simultaneously, the techniques employed to depict molecules are incessantly progressing. Despite the LLM’s recent inception and limited experimentation, the techniques utilized for molecule generation present considerable promise in facilitating the exploration of unexplored realms within biochemical space^11–13^.

Understanding pharmacokinetics and pharmacodynamics is necessary during drug discovery and development. ChatGPT or LLM helps provide information about a particular compound’s pharmacokinetics and pharmacodynamics during the drug discovery and development process. It can provide the toxicity features of drug-like molecules during drug discovery and development^11,14^. AI has already been applied in the phases of a clinical trial of a molecule during the development process and it reduces the clinical trial time^2^. At the same time, AI-enabled ChatGPT might help in clinical trials. Its robust network can significantly decrease clinical trial time^14^. Another parameter studied during drug discovery and development is drug-drug interactions (DDIs). DDIs help us to understand the reaction interface with one or other molecules. Juhi *et al*. have informed us that ChatGPT helps us to understand the DDIs of a molecule. Due to incorrect information by ChatGPT, researchers are talking about the domain-specific ChatGPT. A domain-specific next-generation LLM may support providing accurate information^16^. Researchers are trying to develop domain-specific LLM in the drug discovery and development domain. Recently, DrugChat, a drug discovery domain-specific LLM, was developed and it can produce drug molecular graphs. DrugChat device consists of three essential items: a LLM, an adapter, and a graph neural network^17^. However, ChatGPT or LLM will improve during the future drug discovery and development process, and it will help pharmaceutical companies with more innovative drug discovery and development methods, accelerating the drug development process^15^.

Recently, AI has revolutionized drug discovery. Biotechnology and pharmaceutical companies have discovered over 150 small-molecule drugs using an AI-first approach. At the same time, it has been noted that more than 15 drug molecules have already entered the clinical trials developed by the AI-first approach. Therefore, AI has shown its potential for generating new chemical entities to lead molecule generation with desired properties, minimizing the labor-intensive methods^18^. Therefore, all pharmaceutical and biotechnology companies whose drug discovery and development activity can use AI-enabled ChatGPT’s transformative potential for faster drug discovery and development. However, pharmaceutical and biotechnology companies should set a roadmap for action and AI-based vision and strategy can speed up and more successful drug discovery and development.

## Ethical approval

This article does not require any human/animal subjects to acquire such approval.

## Sources of funding

This study received no specific grant from any funding agency in the public, commercial, or not-for-profit sectors.

## Author contribution

S.P.: investigation, writing – original draft; M.B. and M.A.I.: validation; C.C.: conceptualization, data curation, investigation, writing – original draft, writing – review and editing. All authors critically reviewed and approved the final version of the manuscript.

## Conflicts of interest disclosures

All authors report no conflicts of interest relevant to this article.

## Research registration unique identifying number (UIN)

1. Name of the registry: not applicable.
2. Unique identifying number or registration ID: not applicable.
3. Hyperlink to your specific registration (must be publicly accessible and will be checked): not applicable.

## Guarantor

Md. Aminul Islam, Department of Microbiology, COVID-19 Diagnostic Lab, Noakhali Science and Technology University, Noakhali 3814, Bangladesh. E-mail: aminulmbg@gmail.com

## Provenance and peer review

Not commissioned, internally peer-reviewed.

## Data availability statement

The data in this correspondence article is not sensitive in nature and is accessible in the public domain. The data is therefore available and not of a confidential nature.
